# High Ultrafiltration Rate Is Associated with Increased All-Cause Mortality in Incident Hemodialysis Patients with a High Cardiothoracic Ratio

**DOI:** 10.3390/jpm12122059

**Published:** 2022-12-13

**Authors:** Lii-Jia Yang, Yu-Lin Chao, I-Ching Kuo, Sheng-Wen Niu, Chi-Chih Hung, Yi-Wen Chiu, Jer-Ming Chang

**Affiliations:** 1 Division of Nephrology, Department of Internal Medicine, Kaohsiung Medical University Hospital, Kaohsiung Medical University, Kaohsiung 80708, Taiwan; 2 Department of Internal Medicine, Kaohsiung Municipal CiJin Hospital, Kaohsiung 80544, Taiwan; 3 Department of Internal Medicine, Kaohsiung Municipal Ta-Tung Hospital, Kaohsiung Medical University, Kaohsiung 80145, Taiwan; 4 Regenerative Medicine and Cell Therapy Research Center, Kaohsiung Medical University, Kaohsiung 80708, Taiwan

**Keywords:** ultrafiltration rate, all-cause mortality, cardiothoracic ratio, hemodialysis

## Abstract

A high ultrafiltration rate (UFR) is associated with increased mortality in hemodialysis patients. However, whether a high UFR itself or heart failure with fluid overload followed by a high UFR causes mortality remains unknown. In this study, 2615 incident hemodialysis patients were categorized according to their initial cardiothoracic ratios (CTRs) to assess whether UFR was associated with mortality in patients with high or low CTRs. In total, 1317 patients (50.4%) were women and 1261 (48.2%) were diabetic. During 2246 (1087–3596) days of follow-up, 1247 (47.7%) cases of all-cause mortality were noted. UFR quintiles 4 and 5 were associated with higher risks of all-cause mortality than UFR quintile 2 in fully adjusted Cox regression analysis. As the UFR increased by 1 mL/kg/h, the risk of all-cause mortality increased 1.6%. Subgroup analysis revealed that in UFR quintile 5, hazard ratios (HRs) for all-cause mortality were 1.91, 1.48, 1.22, and 1.10 for CTRs of >55%, 50–55%, 45–50%, and <45%, respectively. HRs for all-cause mortality were higher in women and patients with high body weight. Thus, high UFRs may be associated with increased all-cause mortality in incident hemodialysis patients with a high CTR, but not in those with a low CTR.

## 1. Introduction

**Figure 1:**
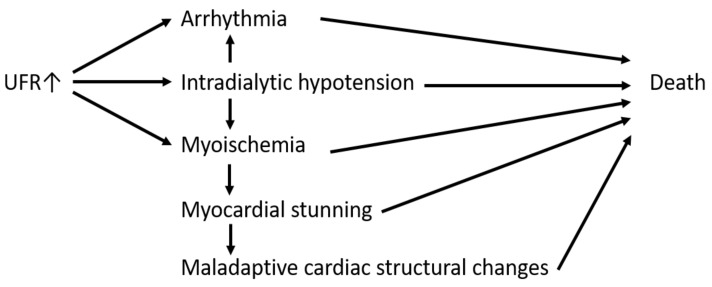
Plausible mechanisms by which high UFR is associated with increased mortality.

Fluid overload is associated with increased mortality in maintenance hemodialysis patients [1]. To mitigate fluid overload, increased fluid removal during hemodialysis is occasionally necessary, resulting in a high ultrafiltration rate (UFR). Rapid fluid removal and subsequent hemodynamic changes during hemodialysis may result in intradialytic hypotension [2,3], arrhythmia [4], maladaptive cardiac structural changes [5,6], myocardial ischemia [7,8], myocardial stunning [9], and even sudden death [10]. In addition to cardiac injury, other forms of end-organ damage, such as brain injury [11] and mesenteric ischemia [12], have been reported. On top of organ damage, rapid fluid removal might cause mortality [13,14,15,16,17]. In the Dialysis Outcomes and Practice Patterns Study (DOPPS), Saran et al. reported that a UFR greater than 10 mL/kg/h was associated with an increased risk of mortality [13]. In a post hoc analysis of the Hemodialysis (HEMO) Study, Flythe et al. demonstrated that a UFR greater than 13 mL/kg/h was significantly associated with increased all-cause mortality and cardiovascular mortality [14]. The plausible mechanism by which a high UFR is associated with increased mortality is depicted in Figure 1. 

Does the association between a high UFR and increased mortality apply to all hemodialysis patients? Flythe et al. reported that a UFR of 10–13 mL/kg/h was associated with increased mortality in patients with congestive heart failure (CHF), but not in those without CHF [14]. Theoretically, hemodialysis patients with CHF should be more intolerant of a high UFR because rapid fluid removal from the intravascular space should be counterbalanced by increasing cardiac output. Since CHF commonly occurs in dialysis patients [18,19], and dialysis patients with CHF have been reported to exhibit higher mortality than those without CHF [18,20], the question becomes: Does high UFR itself or CHF with fluid overload followed by a high UFR cause mortality? If high UFR itself is the cause, we should reduce UFR by decreasing interdialytic weight gain (IDWG) or increasing the dialysis time. If intolerance to a high UFR in patients with CHF is the cause, CHF should be treated.

The diagnosis of CHF is based on symptoms, which may be unreliable in dialysis patients. Echocardiography provides evidence of structural and functional cardiac abnormalities and differentiates between reduced and preserved ejection fractions in the diagnosis of CHF [21]. However, echocardiography is not a routine procedure and is rarely repeated. By contrast, measurement of the cardiothoracic ratio (CTR) by chest radiography is an annual routine assessment in hemodialysis patients in Taiwan under the regulation of the Taiwan Society of Nephrology. Patients with CHF generally have a higher CTR than healthy individuals [22], and a negative correlation between CTR and ejection fraction has been reported [23]. A higher CTR (>0.49) may be a marker of left ventricular hypertrophy (LVH), with a high accuracy of 84.4% [24]. Previous studies have reported that cardiomegaly, assessed by the CTR, is correlated with all-cause mortality and cardiovascular mortality [25,26]. Thus, it seems reasonable to use CTR as a surrogate for CHF.

In brief, CHF is an important confounding factor in the association between UFR and mortality. In this study, we used cardiomegaly, assessed by CTR, as a surrogate for CHF in incident hemodialysis patients to investigate whether a high UFR itself or cardiomegaly followed by a high UFR causes mortality. 

## 2. Patients and Methods 

### 2.1. Participants and Design

This study (the Kaohsiung Hemodialysis Study) was designed as a prospective cohort study to assess the quality of patient care on the basis of the Hemodialysis, Operation, Plan, Executive System developed by the Taiwan Society of Nephrology. In total, 2748 consecutive incident hemodialysis patients who underwent stable dialysis thrice a week for more than 90 days and were aged more than 18 years were recruited from three affiliated hospitals and nine associated hemodialysis clinics of Kaohsiung Medical University, southern Taiwan, between 11 November 2002, and 31 May 2009, and followed until 31 December 2014. Of these, 94 patients were lost to follow-up in less than 6 months, and 39 patients had more than 10% missing data. Thus, the final study population consisted of 2615 incident hemodialysis patients. These patients were divided into 5 quintiles based on the average UFR between the 4th and 9th month after dialysis initiation, with UFR quintile 5 having the highest UFR. This clinical study was conducted in accordance with the Declaration of Helsinki and was approved by the Medical Ethics Committee of Kaohsiung Medical University Hospital.

### 2.2. Measurements

Baseline variables included demographic features (age, sex, and dialysis vintage) and medical history (diabetes mellitus [DM], CHF, ischemic heart disease, hypertension, and cardiovascular disease [CVD]). We collected and averaged examination findings (predialysis and postdialysis body weight [BW]), laboratory data (white blood cell [WBC] count, hemoglobin, creatinine, albumin, glucose, cholesterol, triglyceride, sodium, calcium, phosphate, intact parathyroid hormone, iron saturation, and ferritin), and hemodialysis parameters (UF volume, vascular access, Kt/V, urea reduction ratio, and normalized protein catabolic rate [nPCR]) between the 4th and 9th month after dialysis initiation. The demographic features were obtained from baseline records, and the medical history was obtained through medical chart review. DM and hypertension were assessed by clinical diagnosis. CVD was defined as a clinical diagnosis of CHF, acute or chronic ischemic heart disease, or cerebrovascular disease. Laboratory data were collected monthly; the final data in the analysis were the mean data for 6 months after stable dialysis. Dialysis adequacy was assessed using the single-pool Daugirdas formula Kt/V = −1n([postBUN/preBUN] − 0.008 × t) + ([4 − 3.5 × {postBUN/preBUN}] × UF volume/BW), where K = dialyzer clearance (ml/min), V = urea distribution volume (mL), t = time (min), UF volume = ultrafiltration volume (kg), BW = body weight (kg), and BUN = blood urea nitrogen (mg/dL). A chest radiograph was taken after hemodialysis in the standing posterior–anterior view and within 3–6 months after dialysis initiation. CTR was calculated by dividing the transverse diameter of the heart by the internal diameter of the rib cage.

### 2.3. Outcomes

Mortality, cardiovascular mortality, and cause of death were ascertained by reviewing death certificates using medical charts or the National Death Index. Cardiovascular mortality was defined as the occurrence of death due to ischemic heart disease (the International Classification of Diseases, Ninth Revision, Clinical Modification (ICD-9-CM) codes 410–414), cerebrovascular disease (ICD-9-CM codes 430–438), other forms of heart disease (ICD-9-CM codes 420–429), peripheral arterial occlusion disease (ICD-9-CM codes 443, 441), or hyperkalemia (ICD-9-CM codes 276.7).

### 2.4. Statistical Analysis

Baseline characteristics are expressed as percentages for categorical data, means ± standard deviations for continuous variables with approximately normal distribution, and medians and interquartile ranges for continuous variables with skewed distribution. The differences between groups were verified by using a chi-square test for categorical variables or one-way analysis of variance for continuous variables. The Markov chain Monte Carlo method was used to handle missing covariates (less than 5% missing values in these covariates). The ANOVA and Kruskal–Wallis tests were used to compare variables with normal distribution and skewed distribution among UFR quintiles, respectively. Multivariate linear regression analysis was performed to assess the relationship between UFR and the significant factors listed in Table 1. Cox proportional hazards analysis was performed to assess the relationship between UFR and mortality from the 4th month until the end of the 120th month of hemodialysis or death. Continuous UFR and UFR quintiles were used to assess the linear and non-linear association, respectively [14]. The analysis was initially performed without adjustment and then was fully adjusted by age at dialysis initiation, sex, entry year, DM, hypertension, CHF, stroke, cancer, hepatitis, BW, Kt/V, nPCR, CTR, creatinine, hemoglobin, WBC, albumin, glucose, log-transformed cholesterol, calcium, phosphorus, and iron saturation. Prespecified subgroup analyses were also performed in participants, stratified by sex, age (65 years), DM, hypertension, hemoglobin level (10 g/dL), albumin level (3.5 g/dL), and CTR. Interactions between subgroups were assessed. The final Cox regression model also included significant interactions. A *p* value of <0.05 was considered statistically significant. Statistical analysis was performed using R 4.2.0 software (R Foundation for Statistical Computing, Vienna, Austria) and SPSS version 21.0 for Windows (SPSS Inc., Chicago, IL, USA).

## 3. Results 

### 3.1. Characteristics of Hemodialysis Patients

This study included 2615 incident hemodialysis patients with a mean age of 59.1 ± 14.2 years at dialysis initiation. In total, 50.4% of the patients were women, 48.2% had DM, and 32.5% had CHF (Table 1). The mean UFR was 9.6 ± 3.8 mL/kg/h, and the mean CTR was 49.4 ± 6.5%. 

Patients with a high UFR had a lower age, dry weight, Kt/V, and hemoglobin level; a higher creatinine level, phosphorous level, and UF volume/BW ratio; and a higher prevalence of DM, hypertension, and CHF. UFR quintiles 5 and 1 were associated with a higher prevalence of all-cause mortality and cardiovascular mortality than UFR quintile 2.

### 3.2. Factors Associated with UFR 

The findings of multivariate linear regression analysis (fully adjusted model) of UFR are presented in Table 2. The factors positively associated with UFR were DM, CHF, fasting glucose level, and phosphorus level. The factors negatively associated with UFR were age, female, cancer, hepatitis, and BW. 

### 3.3. Association between UFR and Mortality

During 2246 (1087–3596) days of follow-up, 1247 (47.7%) cases of all-cause mortality and 384 (14.7%) cases of cardiovascular mortality were noted. As shown in Table 3, UFR quintiles 4 and 5 were associated with higher risks of all-cause mortality (hazard ratio [HR]: 1.36, 95% confidence interval [CI]: 1.07–1.74 and HR: 1.38, 95% CI: 1.09–1.74, respectively) than was UFR quintile 2 in fully adjusted Cox regression analysis. As the UFR increased by 1 mL/kg/h, the risk of all-cause mortality also significantly increased (HR: 1.016, 95% CI: 1.003–1.028). UFR quintile 5 was marginally associated with a higher risk of cardiovascular mortality (HR: 1.37, 95% CI: 0.98–1.90) in fully adjusted competing risk Cox regression analysis. 

### 3.4. Echocardiographic Parameters Associated with CTR 

A total of 212 patients in the dialysis centers affiliated with the university received an echocardiography exam during follow-up. The findings of the multivariate linear regression analysis of the echocardiographic parameters and CTR are presented in Supplement Table S1. The factor negatively associated with CTR was mitral annulus early diastolic velocity, a reflect of LV diastolic function.

### 3.5. Subgroup Analysis

**Figure 2:**
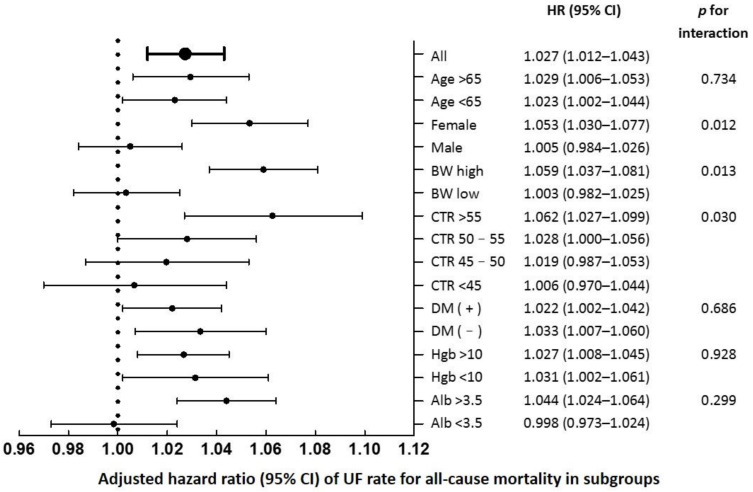
Association between ultrafiltration rate and all-cause mortality across subgroups.

Figure 2 shows the association between UFR and all-cause mortality across subgroups. A linear association between a high UFR and increased all-cause mortality was not consistently noted across subgroups. The association was not significant in patients who were men, had low BW, had CTR < 50%, and had an albumin level < 3.5. As shown in Table 4, compared with UFR quintile 2, UFR quintile 5 was associated with higher mortality in the CTR > 55% and CTR = 50–55% subgroups (HR: 1.91, 95% CI: 1.30–2.82 and HR: 1.48, 95% CI: 1.04–2.12, respectively), but not in the CTR = 45–50% and CTR < 45% subgroups after multivariate adjustment. Moreover, compared with UFR quintile 2, UFR quintile 5 was associated with higher mortality in female patients and patients with high BW (HR: 1.73, 95% CI: 1.31–2.28 and HR: 1.83, 95% CI: 1.38–2.43, respectively), but not in male patients and patients with low BW after multivariate adjustment. 

## 4. Discussion 

In our prospective cohort of 2615 incident hemodialysis patients, we found that a high UFR was linearly associated with a higher risk of all-cause mortality and that the association was affected by CTR, sex, and BW. A high UFR was associated with increased mortality in hemodialysis patients with a high CTR, but not in those with a low CTR.

We found that a high UFR was associated with increased mortality; this finding is consistent with previous findings. In the HEMO Study that was conducted in the United States and involved a high percentage of African Americans (62.6%), a UFR greater than 13 mL/kg/h was associated with increased mortality. In the DOPPS that was conducted in Europe, the United States, and Japan and involved a lower percentage of African American participants (15%), a UFR greater than 10 mL/kg/h was associated with increased mortality. In our study, which involved all Asian participants, UFR quintiles 4 and 5 (with a mean UFR of 11.2 mL/kg/h and 15.0 mL/kg/h, respectively) were associated with increased mortality. Although the cutoff values varied among studies and races, the association between UFR and mortality was consistently noted. 

We also determined that the association between UFR and mortality was affected by CTR. UFR quintile 5 (>12.3 mL/kg/h) was associated with increased mortality only in patients with CTR > 50%, but not in those with CTR < 50%. A previous study reported that a UFR greater than 13 mL/kg/h was associated with increased mortality, regardless of CHF, whereas a UFR of 10–13 mL/kg/h was associated with increased mortality in patients with CHF, but not in those without CHF [14]. Adequate activation of the sympathetic nervous system during hemodialysis is crucial to maintain blood pressure by increasing the cardiac output and peripheral vascular resistance. However, in patients with CHF, the cardiac output is impaired and cannot be appropriately increased to match the metabolic demands under stress, regardless of the ejection fraction [27,28]. LVH, a precursor of CHF, causes decreased ventricular distensibility, making the ventricle more sensitive to volume changes. Consequently, even a small degree of UF-induced hypovolemia may lead to a decrease in filling pressure and cause systemic hypotension [29]. Moreover, hemodialysis has been reported to be associated with a pronounced decrease in myocardial blood flow, causing myocardial ischemia and worsened cardiac dysfunction during dialysis [30]. A combination of CHF and hemodialysis may increase the occurrence of intradialytic hypotension, which has been reported to be an independent risk factor for mortality in hemodialysis patients [2]. This may partly explain why the association between high UFR and mortality was noted only in patients with CTR > 50%, but not in those with CTR < 50%. Because the association between a high UFR and mortality was noted in patients with a high CTR, but not in those with a low CTR, CHF with fluid overload followed by a high UFR rather than a high UFR itself can cause mortality. Consequently, for hemodialysis patients with cardiomegaly, direct therapy for CHF should be applied in addition to adequate control of IDWG and a longer session time. 

We found that sex influenced the association between UFR and mortality. A high UFR was associated with increased mortality in women, but not in men. Similarly, a previous study reported a slight difference in this association between men and women [31]. The prevalence of HF is higher in women than in men globally (9.16 versus 7.69 per million inhabitants) [32]. Women have smaller LV chambers and greater wall thickness than men after adjustment for body size and age [33]. Compared with men, women have lower diastolic compliance and a higher tendency of concentric ventricular remodeling [34,35]. Moreover, women tend to have a higher prevalence of endothelial dysfunction, arterial stiffness, and coronary microvascular dysfunction [35]. Women also exhibit a stronger immune response than men [36], which may lead to enhanced inflammatory reactions in the female myocardium. Because of these structural and functional differences, women are the prime candidates for the development of HF with preserved ejection fraction. Our unpublished data also revealed a higher CTR in women, which is in accordance with our major findings. 

Finally, we found that the association between UFR and mortality was affected by BW; this finding is consistent with previous findings. A high UFR was more strongly associated with increased mortality among patients with higher BW than in those with lower BW [16,31]. The association between obesity (body mass index > 30) and increased mortality has been reported in a previous study [37]. Obesity is associated with increased cardiovascular complications through multiple processes, such as inflammation, insulin resistance, endothelial dysfunction, coronary calcification, and activation of the coagulation and sympathetic nervous systems [38]. Patients with higher BW may have more cardiovascular risk factors and may, therefore, be more vulnerable to complications related to a high UFR, such as intradialytic hypotension and myocardial stunning. However, the mechanism underlying the effect of BW on the association between UFR and mortality warrants further investigation. 

### Limitations 

The limitations of our study should be acknowledged. First, our study is observational. Thus, some residual confounding may have existed, and the causal relationship between a high UFR and mortality could not be confirmed. Although we adjusted multiple comorbidities, cardiomyopathy was not taken into consideration. Dilated cardiomyopathy may lead to heart failure [39], and hypertrophic cardiomyopathy could lead to syncope, life-threatening arrhythmic events, and even sudden cardiac death [40]. Both could be confounding factors in the research of mortality in hemodialysis patients. No record or adjustment for cardiomyopathies may lead to biases. Second, it is difficult to fully assess cardiac condition without functional or structural data from echocardiography. Since echocardiography was not mandatory for hemodialysis patient under the regulation of the Taiwan Society of Nephrology, only a small proportion of patients received echocardiography in the current study. Without adjusting baseline cardiac status, some biases may exist. Third, we did not measure urine volume. Hemodialysis patients with more residual urine output may have less volume overload and better survival [41]. However, in our previous report, patients with chronic kidney disease in southern Taiwan started dialysis late, with a mean estimated glomerular filtration rate of 5 mL/min/1.73 m^2^ and very little residual urine [42]. Fourth, we did not record IDWG, which could be different from UF. At steady state, UF is equivalent to IDWG, and thus it might be reasonable to assume the effect of IDWG on clinical outcome was positively correlated with UFR. However, if patients with high IDWG could not tolerate the large fluid being removed, the UF may be less than IDWG, and thus these patients might be inappropriately allocated to the low UFR group. This mismatch could lead to biases. To minimize the impact of a single mismatch, we classified patients on the basis of the average UFR between the 4th and 9th month after dialysis initiation. Through multiple adjustment of dry BW, the UF would be close to IDWG. Fifth, we used observed UFR and not prescribed UFR. The prescribed UFR could be higher than the observed UFR and could be more deleterious. Sixth, the blood pressure and medication data of the patients were not recorded. 

## 5. Conclusions

In incident hemodialysis patients, a high UFR is associated with a higher risk of all-cause mortality. This association remains consistent in patients with CTR > 50%, but not in those with CTR < 50%, indicating that heart failure with fluid overload followed by a high UFR, rather than a high UFR itself, may be the cause of mortality. 

## Figures and Tables

**Table 1:** Clinical characteristics and outcomes of incident hemodialysis patients stratified into five quintiles based on baseline ultrafiltration rate.

Variables		Ultrafiltration Rate					
	Total	Q1	Q2	Q3	Q4	Q5	*p* (Anova)
UFR (mL/kg/h)		<6.6	6.6–8.6	8.6–10.3	10.3–12.3	>12.3	
No. of patients	2615	523 (20.0%)	523 (20.0%)	523 (20.0%)	523 (20.0%)	523 (20.0%)	
Age at dialysis (year)	59.1 (14.2)	64.0 (12.9)	60.2 (13.8)	58.6 (13.6)	57.7 (14.0)	54.9 (15.0)	<0.001
Gender (female)	1317 (50.4%)	263 (50.3%)	257 (49.1%)	259 (49.5%)	270 (51.6%)	268 (51.2%)	0.916
**Comorbidities**							
Diabetes mellitus	1261 (48.2%)	225 (43.0%)	218 (41.7%)	264 (50.5%)	286 (54.7%)	268 (51.2%)	<0.001
Hypertension	1831 (70.0%)	340 (65.0%)	367 (70.2%)	391 (74.8%)	375 (71.7%)	358 (68.5%)	0.010
Heart failure	850 (32.5%)	143 (27.3%)	166 (31.7%)	168 (32.1%)	181 (34.6%)	192 (36.7%)	0.018
Stroke	194 (7.4%)	33 (6.3%)	41 (7.8%)	43 (8.2%)	45 (8.6%)	32 (6.1%)	0.417
Cancer	161 (6.2%)	50 (9.6%)	33 (6.3%)	37 (7.1%)	24 (4.6%)	17 (3.3%)	<0.001
**Dialysis status**							
Dry body weight (kg)	56.8 (11.7)	57.7 (11.6)	58.3 (12.5)	58.1 (11.9)	56.4 (11.5)	52.9 (9.5)	<0.001
Kt/V (Gotch)	1.30 (0.23)	1.30 (0.23)	1.30 (0.24)	1.30 (0.24)	1.30 (0.22)	1.30 (0.23)	0.995
Kt/V < 1.4	1805 (69.0%)	325 (62.1%)	347 (66.3%)	364 (69.6%)	374 (71.5%)	395 (75.5%)	<0.001
nPCR	1.2 (0.3)	1.1 (0.3)	1.2 (0.3)	1.1 (0.3)	1.2 (0.3)	1.2 (0.3)	0.006
UFR (mL/kg/h)	9.6 (3.8)	4.8 (1.5)	7.6 (0.6)	9.4 (0.5)	11.2 (0.6)	15.0 (3.1)	<0.001
UF/BW ratio (%)	3.8 (1.5)	1.9 (0.6)	3.1 (0.2)	3.8 (0.2)	4.5 (0.2)	5.9 (1.3)	<0.001
Dialysis length (min)	239.0 (6.8)	239.2 (5.8)	239.7 (6.0)	239.1 (7.1)	239.4 (4.4)	237.5 (9.3)	<0.001
CTR (%)	50.3 (6.5)	50.9 (6.3)	50.2 (6.4)	50.0 (6.4)	50.0 (6.8)	50.5 (6.5)	0.128
**Laboratory Results**							
WBC (×1000/μL)	7.0 (2.3)	7.2 (2.7)	6.9 (2.1)	6.9 (2.1)	7.0 (2.2)	6.9 (2.2)	0.300
Hemoglobin (g/dL)	9.9 (1.2)	10.0 (1.3)	9.9 (1.2)	10.0 (1.2)	9.8 (1.2)	9.6 (1.2)	<0.001
Albumin (gm/dL)	3.7 (0.4)	3.7 (0.4)	3.8 (0.4)	3.8 (0.4)	3.7 (0.4)	3.7 (0.4)	0.013
Creatinine (mg/dL)	9.2 (2.8)	8.8 (2.7)	9.5 (2.9)	9.5 (2.7)	9.2 (2.9)	9.3 (3.0)	<0.001
Glucose [AC] (mg/dL)	136.4 (60.8)	126.2 (50.8)	130.6 (53.9)	135.5 (60.6)	142.6 (63.8)	147.2 (70.5)	<0.001
Cholesterol (mg/dL)	184 (156–215)	181 (152–211)	186 (158–214)	182 (157–215)	186 (155–220)	185 (156–216)	0.238
Triglyceride (mg/dL)	142 (99–210)	143 (102–206)	145 (99–224)	141 (98–207)	146 (97–226)	136 (100–196)	0.252
Sodium (meq/L)	138.1 (3.5)	138.8 (3.4)	138.7 (3.2)	137.9 (3.4)	137.8 (3.5)	137.6 (3.7)	<0.001
Calcium (mg/dL)	9.3 (0.8)	9.4 (0.8)	9.3 (0.7)	9.3 (0.8)	9.3 (0.8)	9.2 (0.8)	0.001
Phosphate (mg/dL)	5.0 (1.2)	4.6 (1.2)	4.9 (1.1)	5.0 (1.2)	5.1 (1.3)	5.2 (1.3)	<0.001
intact-PTH (pg/mL)	176 (64–359)	179 (60–360)	176 (59–352)	173 (67–351)	167 (66–339)	179 (67–392)	0.944
Iron saturation (%)	32.4 (20.5)	31.8 (15.6)	32.5 (17.1)	32.4 (14.4)	33.6 (25.8)	31.6 (26.5)	0.513
Ferritin (ng/mL)	519 (318–798)	534 (345–803)	528 (327–805)	507 (336–764)	536 (282–830)	483 (287–797)	0.384
**Outcomes**							
Follow-up days	2246 (1087–3596)	2144 (985–3480)	2378 (1187–3585)	2394 (1171–3708)	2090 (1017–3391)	2268 (1049–3732)	0.068
Mortality	1247 (47.7%)	264 (50.5%)	230 (44.0%)	232 (44.4%)	253 (48.4%)	268 (51.2%)	0.047
CV mortality	384 (14.7%)	90 (17.2%)	66 (12.6%)	70 (13.4%)	69 (13.2%)	89 (17.0%)	0.035

Abbreviations: UFR, ultrafiltration rate; BW, body weight; nPCR, normalized protein catabolic rate; CTR, cardiothoracic ratio; HD, hemodialysis; CV, cardiovascular; WBC, white blood cell; Glucose [AC], fasting glucose; PTH, parathyroid hormone.

**Table 2:** Multivariate linear regression analysis of ultrafiltration rate.

Variables	β	95% CI of β	*p*-Value
Age at dialysis	−0.069	−0.081 to −0.057	<0.001
Gender (female vs. male)	−0.418	−0.771 to −0.066	0.020
Entry year (late vs. early)	−0.289	−0.611 to 0.034	0.079
Diabetes	0.834	0.496 to 1.173	<0.001
Hypertension	0.205	−0.118 to 0.527	0.214
Heart failure	0.383	0.064 to 0.701	0.018
Stroke	0.240	−0.296 to 0.777	0.380
Cancer	−0.577	−1.146 to −0.008	0.047
Hepatitis	−0.512	−0.909 to −0.116	0.011
Hemoglobin (g/dL)	−0.052	−0.180 to 0.076	0.424
Dry body weight (kg)	−0.089	−0.104 to −0.073	<0.001
Kt/V (Gotch)	−0.282	−1.125 to 0.560	0.512
nPCR	0.057	−0.500 to 0.614	0.840
Cardiothoracic ratio (%)	−0.004	−0.028 to 0.019	0.716
Albumin (gm/dL)	−0.278	−0.721 to 0.165	0.219
WBC (×1000/μL)	−0.060	−0.130 to 0.009	0.088
Cholesterol	−0.179	−1.654 to 1.297	0.812
Glucose (mg/dL)	0.008	0.005 to 0.010	<0.001
Phosphate (mg/dL)	0.502	0.366 to 0.637	<0.001
Iron saturation	0.003	−0.004 to 0.009	0.461

Abbreviations: DM, diabetes mellitus; HTN, hypertension; CHF, congestive heart failure; BW, body weight; nPCR, normalized protein catabolic rate; WBC, white blood cell.

**Table 3:** Association between ultrafiltration rate and clinical outcomes.

	Ultrafiltration Rate (1 mL/kg/h)	Ultrafiltration Rate				
		Q1	Q2	Q3	Q4	Q5
HR for all-cause mortality						
Unadjusted	1.007 (0.991–1.022)	1.23 (1.03–1.46)	1 (reference)	1.00 (0.84–1.20)	1.18 (0.99–1.41)	1.19 (1.00–1.42)
Model 1 ^a^	1.036 (1.021–1.050) *	0.99 (0.83–1.19)	1 (reference)	1.03 (0.86–1.24)	1.24 (1.03–1.48) *	1.50 (1.25–1.79) *
Model 2 ^b^	1.027 (1.012–1.043) *	1.03 (0.86–1.23)	1 (reference)	1.13 (0.94–1.36)	1.24 (1.03–1.49) *	1.44 (1.20–1.74) *
Model 3 ^c^	1.016 (1.003–1.028) *	1.22 (0.98–1.53)	1 (reference)	1.21 (0.95–1.54)	1.36 (1.07–1.74) *	1.38 (1.09–1.74) *
HR for CV mortality						
Unadjusted	1.000 (0.972–1.028)	1.40 (1.02–1.92)	1 (reference)	1.07 (0.76–1.49)	1.07 (0.76–1.50)	1.35 (0.98–1.86)
Model 1 ^a^	1.002 (0.973–1.030)	1.35 (0.98–1.85)	1 (reference)	1.08 (0.79–1.49)	1.07 (0.75–1.51)	1.36 (0.98–1.86)
Model 2 ^b^	1.003 (0.975–1.032)	1.27 (0.92–1.76)	1 (reference)	1.10 (0.78–1.55)	1.00 (0.71–1.41)	1.38 (0.99–1.92)
Model 3 ^c^	1.003 (0.974–1.033)	1.29 (0.93–1.78)	1 (reference)	1.21 (0.86–1.71)	1.14 (0.80–1.62)	1.37 (0.98–1.90)

^a^ Model 1: adjusted by age at dialysis initiation, sex, entry year, DM, HTN, CHF, stroke, cancer, and hepatitis. ^b^ Model 2: adjusted by age at dialysis initiation, sex, entry year, DM, HTN, CHF, stroke, cancer, hepatitis, BW, Kt/V, nPCR, CTR, WBC, hemoglobin, glucose, cholesterol, albumin, calcium, phosphate, and iron saturation. ^c^ Model 3: adjusted by age at dialysis initiation, sex, entry year, DM, HTN, CHF, stroke, cancer, hepatitis, BW, Kt/V, nPCR, CTR, WBC, hemoglobin, glucose, cholesterol, albumin, calcium, phosphate, iron saturation, UFR*CTR, UFR*sex, and UFR*BW. Abbreviations: DM, diabetes mellitus; HTN, hypertension; CHF, congestive heart failure; BW, body weight; nPCR, normalized protein catabolic rate; CTR, cardiothoracic ratio. * indicates *p* < 0.05.

**Table 4:** Hazard ratios for all-cause mortality according to CTR, sex, and dry body weight.

	Ultrafiltration Rate					
	Q1	Q2	Q3	Q4	Q5	*p* for Interaction
CTR ^a^						0.026
CTR < 45%	0.82 (0.53–1.29)	1 (reference)	0.73 (0.47–1.16)	1.07 (0.70–1.65)	1.10 (0.70–1.75)	
CTR 45–50%	0.99 (0.69–1.41)	1 (reference)	1.10 (0.78–1.54)	1.10 (0.77–1.59)	1.22 (0.85–1.75)	
CTR 50–55%	1.00 (0.72–1.38)	1 (reference)	1.31 (0.93–1.85)	1.20 (0.84–1.71)	1.48 (1.04–2.12)	
CTR > 55%	1.18 (0.82–1.71)	1 (reference)	1.17 (0.79–1.75)	1.39 (0.96–2.04)	1.91 (1.30–2.82)	
Gender ^b^						0.071
Female	0.96 (0.75–1.25)	1 (reference)	1.15 (0.88–1.50)	1.20 (0.92–1.57)	1.73 (1.31–2.28)	
Male	1.08 (0.84–1.40)	1 (reference)	1.05 (0.81–1.37)	1.25 (0.96–1.62)	1.21 (0.93–1.57)	
Dry BW ^c^						0.095
High ^d^	0.90 (0.68–1.17)	1 (reference)	1.21 (0.93–1.57)	1.50 (1.14–1.96)	1.83 (1.38–2.43)	
Low	1.08 (0.84–1.38)	1 (reference)	0.98 (0.75–1.28)	1.00 (0.77–1.29)	1.18 (0.92–1.52)	

Abbreviations: DM, diabetes mellitus; HTN, hypertension; CHF, congestive heart failure; BW, body weight; nPCR, normalized protein catabolic rate; CTR, cardiothoracic ratio. *p* < 0.05 in all three subgroups. ^a^ adjusted by age at dialysis initiation, sex, entry year, DM, HTN, CHF, stroke, cancer, hepatitis, BW, Kt/V, and nPCR. ^b^ adjusted by age at dialysis initiation, entry year, DM, HTN, CHF, stroke, cancer, hepatitis, BW, Kt/V, nPCR, and CTR. ^c^ adjusted by age at dialysis initiation, sex, entry year, DM, HTN, CHF, stroke, cancer, hepatitis, Kt/V, nPCR, and CTR. ^d^ High BW was defined as BW higher than mean BW, and low BW was defined as BW lower than mean BW.

## Data Availability

The data presented in this study are available on request from the corresponding author (CCH).

## Supplementary Materials

The following supporting information can be downloaded at: https://www.mdpi.com/article/10.3390/jpm12122059/s1, Table S1: Linear regression analysis of echocardiographic parameters and CTR.
