# Supplementary material for: High Ultrafiltration Rate Is Associated with Increased All-Cause Mortality in Incident Hemodialysis Patients with a High Cardiothoracic Ratio
Source: J Pers Med. 2022 Dec 13;12(12):2059. doi: 10.3390/jpm12122059 (PMC9786000; doi:10.3390/jpm12122059)
Supplement: Supplementary file 1 [file jpm-12-02059-s001.zip › jpm-1949376-supplementary.pdf]

Table S1. Linear regression analysis of echocardiographic parameters and CTR

Variables	Univariate		Multivariate	
	Unstandardized coefficient β (95% CI)	*p*-value-	Unstandardized coefficient β (95% CI)	*p*-value-
LA diameter (per 1 cm)	1.531 (0.210 – 2.851)	0.023	0.295 (-1.339 – 1.929)	0.722
LVIDd (per 1 cm)	-0.892 (-2.210 – 0.426)	0.183	–	–
LVPWd (per 1 cm)	3.017 (-1.854 – 7.889)	0.223	–	–
LVEF (per 1 %)	-0.033 (-0.123– 0.056)	0.461	–	–
LVFS (per 1%)	-0.042 (-0.161 – 0.077)	0.489	–	–
Early mitral valve flow velocity (E) (per 1 cm/s)	0.023 (-0.007 – 0.052)	0.127	–	–
Mitral annulus early diastolic velocity (E') (per 1 cm/s)	-0.898 (-1.266 – -0.530)	< 0.001	-0.877 (-1.354 – -0.401)	< 0.001
Deceleration time of E wave (per 1 ms)	0.001 (-0.014 – 0.015)	0.901	–	–
RWT (per 1 unit)	7.152 (-1.968 – 16.271)	0.124	–	–
E/E'(per 1 unit)	0.142 (0.049 – 0.235)	0.003	-0.016 (-0.136 – 0.104)	0.789
LA volume (per 1 mL)	-0.007 (-0.034 – 0.020)	0.620	–	–
LAVI (per 1 mL/m^2)	0.096 (0.020 – 0.173)	0.014	0.050 (-0.050 – 0.150)	0.323
LVMI (per 1 g/m^2)	0.023 (0.002 – 0.044)	0.034	0.002 (-0.022 – 0.027)	0.846

Abbreviations: CTR, cardiothoracic ratio; LA, left atrial; LVIDd, left ventricular internal diameter at end diastole; LVPWd, left ventricular posterior wall at end diastole; LVEF, left ventricular ejection fraction; LVFS, left ventricular fractional shortening; RWT, relative wall thickness; LAVI, left atrial volume index; LVMI, left ventricular mass index.
